# Supplementary material for: Freeze-Drying of a Capsid Virus-like Particle-Based Platform Allows Stable Storage of Vaccines at Ambient Temperature
Source: Pharmaceutics. 2022 Jun 18;14(6):1301. doi: 10.3390/pharmaceutics14061301 (PMC9229831; doi:10.3390/pharmaceutics14061301)
Supplement: Supplementary file 1 [file pharmaceutics-14-01301-s001.zip › pharmaceutics-1756371-supplementary.pdf]

## Supplementary Material:

# Freeze-drying of a capsid virus-like particle based platform allows stable storage of vaccines at ambient temperature

Kara-Lee Aves, Christoph M. Janitzek, Cyrielle E. Fougeroux, Thor G. Theander, Adam F. Sander*

* Correspondence: asander@sund.ku.dk

**Supplementary Table S1. Cake consistency and the ease of reconstitution of freeze-dried cVLPs under different buffer conditions.** Green indicates the freeze-dried material was in the form of a homogenous powder (i.e. cake solid), which could be reconstituted back into solution with little difficulty. Red indicates the freeze-dried material was hard and crystalline in consistency (i.e. collapsed solid) and required an extended incubation or more vigorous mixing during reconstitution. Yellow indicates an intermediate situation, where the freeze-dried material was in the form of a powder but required extensive mixing during reconstitution.

| | | Stabilizing Agent | | | | |
|---|---|---|---|---|---|---|
| Sample | Buffer | No Stabilizer | +5% Trehaolse | +5% Sucrose | +5% Mannitol | +5% Sucrose +0.005% Tween 20 |
| Catcher.cVLP | 20mM Sodium Phosphate, pH 7.4 | Green | Green | Green | Green | Green |
| Catcher.cVLP | 15mM Tris, 200mM NaCl, pH 8.5 | Green | Green | Red | Green | Red |
| Tag.cVLP | 20mM Sodium Phosphate, pH 7.4 | Yellow | Green | Green | Green | Green |
| Tag.cVLP | 15mM Tris, 200mM NaCl, pH 8.5 | Yellow | Red | Red | Green | Red |

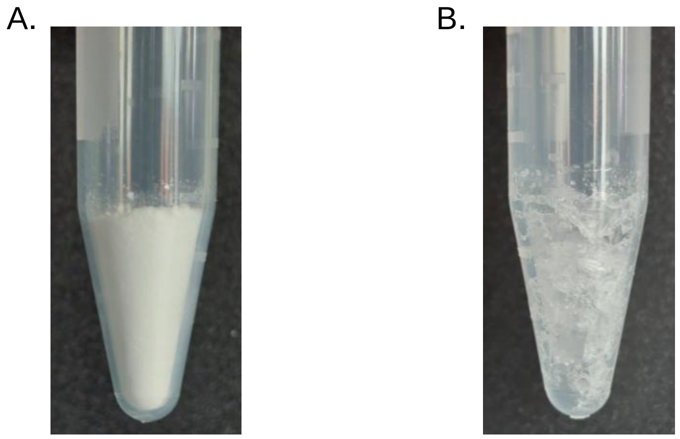


**Supplementary Figure S1. Representative examples of the freeze-dried cake consistence** showing (a) a cake solid (Catcher.cVLP 20mM sodium phosphate buffer + 5% mannitol) and (b) a collapsed crystalline solid (Catcher.cVLP 15mM Tris buffer + 5% sucrose).

**Supplementary Table S2. Summary of the physical properties of reconstituted Catcher.cVLP following freeze-drying under different buffer and excipient conditions and storage for 2 months at ambient temperature or 37℃.** Physical properties of the FD Catcher.cVLP are compared to the non-FD (-80℃) reference sample and are summarized from Figure 1 and S4. Green indicated that the property is maintained in the FD sample. Red indicates that freeze-drying has a negatively effect on the measured physical property. White indicates that the assay was not performed.

| Buffer | Excipient | Storage temperature | Particle diameter | Thermal stability | Particle aggregation | Encapsulated RNA | Particle integrity | Conjugation to Tagged antigen |
|---|---|---|---|---|---|---|---|---|
| 20mM Sodium Phosphate, pH 7.4 | No stabilizer | AT | Red | Green | Green | Green | Green | Green |
| 20mM Sodium Phosphate, pH 7.4 | No stabilizer | 37˚C | Red | Red | Green | Green | Red | Green |
| 20mM Sodium Phosphate, pH 7.4 | 5% trehalose | AT | Green | Green | Green | Green | Green | Green |
| 20mM Sodium Phosphate, pH 7.4 | 5% trehalose | 37˚C | Green | Green | Green | Green | Green | Green |
| 20mM Sodium Phosphate, pH 7.4 | 5% sucrose + 0.005% T20 | AT | Green | Green | Green | Green | Green | Green |
| 20mM Sodium Phosphate, pH 7.4 | 5% sucrose + 0.005% T20 | 37˚C | Green | Green | Green | Green | Green | Green |
| 15mM Tris, 200mM NaCl, pH 8.5 | No stabilizer | AT | Red | | Red | Green | Red | Green |
| 15mM Tris, 200mM NaCl, pH 8.5 | No stabilizer | 37˚C | Red | | Red | Green | Red | Green |
| 15mM Tris, 200mM NaCl, pH 8.5 | 5% trehalose | AT | Green | | Green | Green | Green | Green |
| 15mM Tris, 200mM NaCl, pH 8.5 | 5% trehalose | 37˚C | Green | | Green | Green | Green | Green |
| 15mM Tris, 200mM NaCl, pH 8.5 | 5% sucrose + 0.005% T20 | AT | Green | | Green | Green | Green | Green |
| 15mM Tris, 200mM NaCl, pH 8.5 | 5% sucrose + 0.005% T20 | 37˚C | Red | | Red | Green | Red | Green |

**Supplementary Table S3. Summary of the physical properties of reconstituted Tag.cVLP following freeze-drying under different buffer and excipient conditions and storage for 2 months at ambient temperature or 37˚C**. Physical properties of the FD Tag.cVLP are compared to the non-FD (-80˚C) reference sample and are summarized from Figure S7 and S8. Green indicated that the property is maintained in the FD sample. Red indicates that freeze-drying has a negatively effect on the measured physical property. White indicates that the assay was not performed.

| Buffer | Excipient | Storage temperature | Particle diameter | Thermal stability | Particle aggregation | Encapsulated RNA | Particle integrity | Conjugation to Tagged antigen |
|---|---|---|---|---|---|---|---|---|
| 20mM Sodium Phosphate, pH 7.4 | No stabilizer | AT | Red | Red | Red | Green | Red | Green |
| 20mM Sodium Phosphate, pH 7.4 | No stabilizer | 37˚C | Red | Red | Red | Green | Red | Green |
| 20mM Sodium Phosphate, pH 7.4 | 5% trehalose | AT | Red | Green | Green | Green | Green | Green |
| 20mM Sodium Phosphate, pH 7.4 | 5% trehalose | 37˚C | Red | Green | Green | Green | Green | Green |
| 20mM Sodium Phosphate, pH 7.4 | 5% sucrose + 0.005% T20 | AT | Green | Green | Green | Green | Green | Green |
| 20mM Sodium Phosphate, pH 7.4 | 5% sucrose + 0.005% T20 | 37˚C | Green | Green | Green | Green | Green | Green |
| 15mM Tris, 200mM NaCl, pH 8.5 | No stabilizer | AT | Red | White | Red | Green | Red | Green |
| 15mM Tris, 200mM NaCl, pH 8.5 | No stabilizer | 37˚C | Red | White | Red | Green | Red | Green |
| 15mM Tris, 200mM NaCl, pH 8.5 | 5% trehalose | AT | Green | White | Green | Green | Green | Green |
| 15mM Tris, 200mM NaCl, pH 8.5 | 5% trehalose | 37˚C | Green | White | Green | Green | Green | Green |
| 15mM Tris, 200mM NaCl, pH 8.5 | 5% sucrose + 0.005% T20 | AT | Red | White | Red | Green | Green | Green |
| 15mM Tris, 200mM NaCl, pH 8.5 | 5% sucrose + 0.005% T20 | 37˚C | Red | White | Red | Green | Red | Green |

**Supplementary Table S4. Quantitative dynamic-Light scattering results of freeze-dried Catcher-cVLP, $HA_{stem}$-cVLP and RBD-cVLP.** The hydrodynamic diameter, % polydispersity and % intensity of the main peak population of particles from the representative DLS results presented in the main article figures were quantified using Dynamics software. Results are representative of measurements run in duplicate or triplicate.

| Sample | Condition | Figure | Hydrodynamic diameter (nm) | Polydispersity (%) | Intensity (%) |
|---|---|---|---|---|---|
| Catcher-cVLP | Non-frozen | 1B | 43.6 | 13.82 | 98.7 |
| Catcher-cVLP | Non-FD (-80 ˚C) | 1B | 45.6 | 11.75 | 87.2 |
| Catcher-cVLP No stabilizer | FD-AT | 1B | 77.4 | 32.26 | 94.1 |
| Catcher-cVLP No stabilizer | FD-37˚C | 1B | 100.6 | 42.7 | 97.0 |
| Catcher-cVLP 5% Trehalose | FD-AT | 1B | 49.0 | 23.7 | 100 |
| Catcher-cVLP 5% Trehalose | FD-37˚C | 1B | 38.8 | 14.03 | 73.8 |
| Catcher-cVLP 5% Sucrose + 0.005% T20 | FD-AT | 1B | 48.8 | 24.77 | 89.5 |
| Catcher-cVLP 5% Sucrose + 0.005% T20 | FD-37˚C | 1B | 47.0 | 29.80 | 93.0 |
| $HA_{stem}$-cVLP | Non-FD (-80 ˚C) | 2D | 58.0 | 14.08 | 100 |
| $HA_{stem}$-cVLP | FD-AT | 2D | 60.0 | 12.70 | 95.6 |
| $HA_{stem}$-cVLP | FD-37˚C | 2D | 58.8 | 13.97 | 97.3 |
| RBD-cVLP | Non-FD (-80 ˚C) | 3D | 52.4 | 17.83 | 96.7 |
| RBD-cVLP | FD-AT | 3D | 50.8 | 11.06 | 93.7 |
| RBD-cVLP | FD-37˚C | 3D | 52.8 | 13.51 | 91.2 |

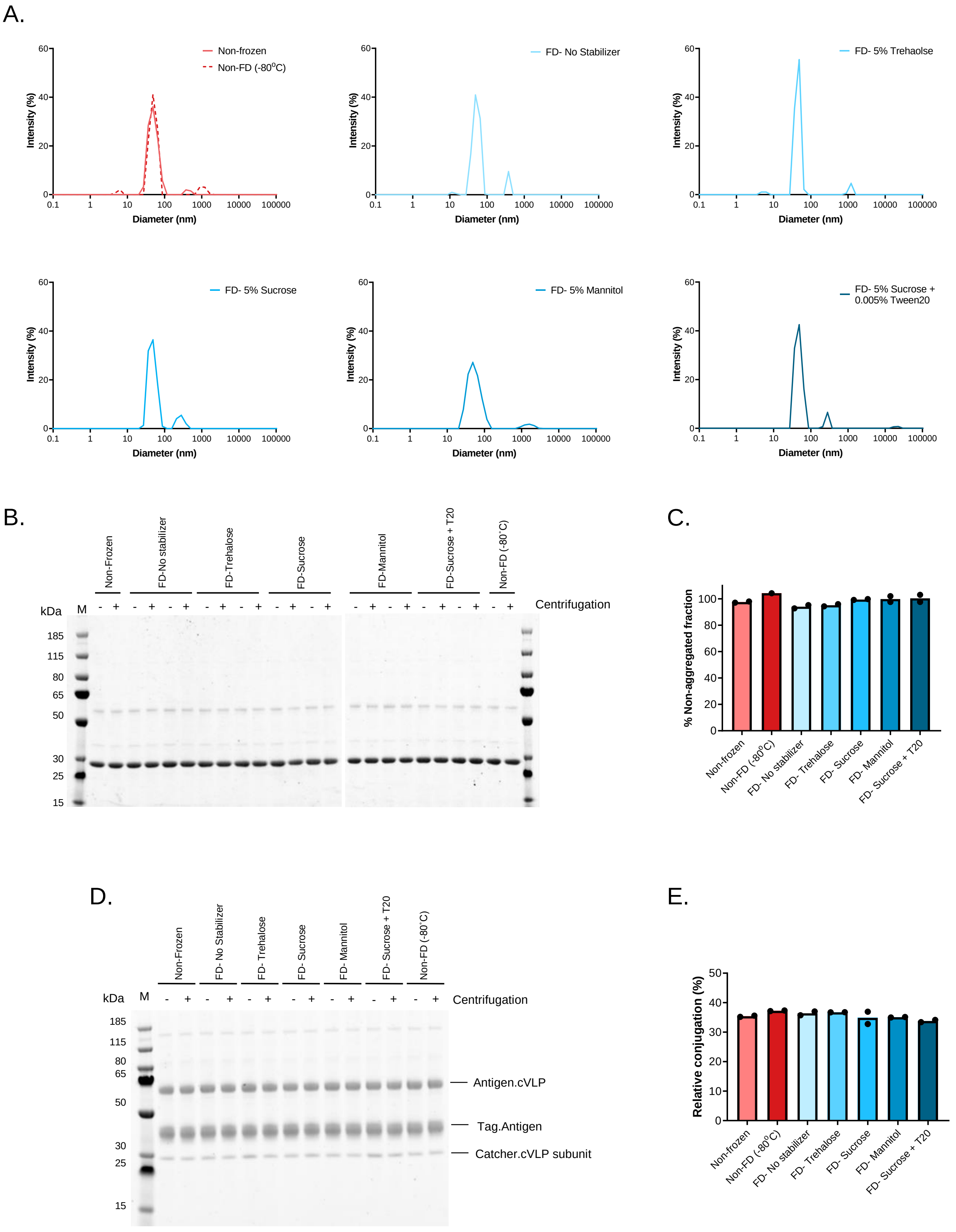


**Supplementary Figure S2. Effect of excipients on the stability of freeze-dried Catcher-cVLPs in a sodium phosphate buffer (pH 7.4).** Catcher-cVLPs formulated in a sodium phosphate buffer (pH 7.4) with no stabilizing agent, 5% trehalose, 5% sucrose, 5% mannitol or 5% sucrose + 0.005% Tween20 were freeze-dried and reconstituted one day later. The stability of freeze-

dried particles was compared to a non-frozen or a frozen non-freeze dried (Non-FD (-80˚C)) reference sample. (a) DLS analysis. (b) Reduced SDS-PAGE analysis of freeze-dried Catcher-cVLPs before (-) or after (+) centrifugation (2 min at 16,000 g). (c) Quantification (by densitometric analysis of SDS-PAGE) of the relative amount (%) of reconstituted Catcher-cVLP, which remain in suspension after centrifugation. (d) Representative SDS-PAGE analysis of the conjugation reaction between freeze-dried Catcher-cVLPs with a tagged antigen. Samples were taken before (-) and after (+) centrifugation of the coupling mixture. Tag:Catcher conjugation results in the formation of an antigen-cVLP coupling band at 60 kDa. (e) Quantification of the antigen-cVLP coupling band from the conjugation reaction of Catcher-cVLPs with a tag-antigen. Results show the mean of samples performed in duplicate.

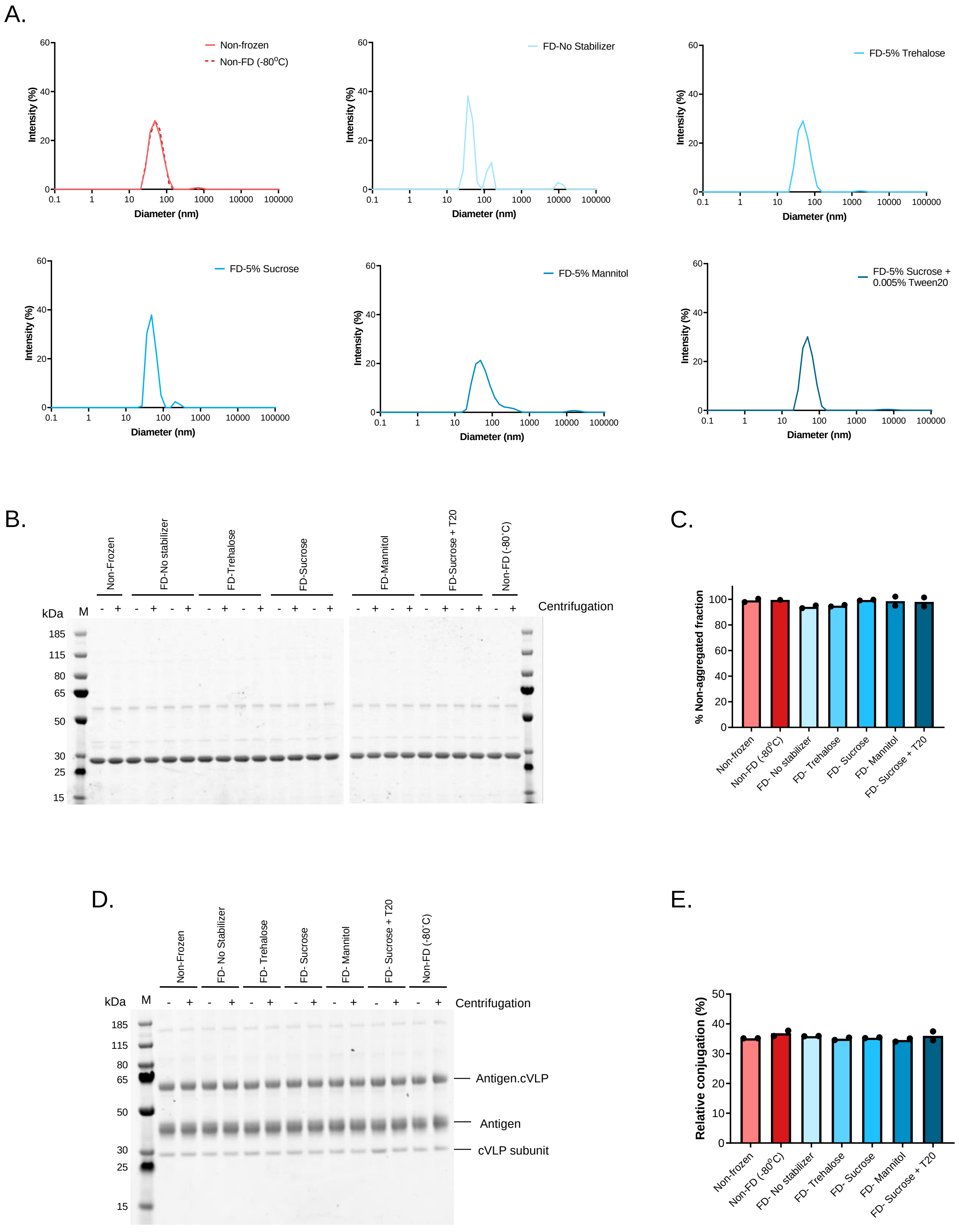


**Supplementary Figure S3. Effect of excipients on the stability of freeze-dried Catcher-cVLPs in a Tris buffer (pH 8.5).** Catcher-cVLPs formulated in a Tris buffer (pH 8.5) with no stabilizing agent, 5% trehalose, 5% sucrose, 5% mannitol or 5% sucrose + 0.005% Tween20 were freeze-

dried and subsequently reconstituted one day later. The stability of freeze-dried particles was compared to a non-frozen or a frozen non-freeze dried (Non-FD (-80˚C)) reference sample. (a) DLS analysis. (b) Reduced SDS-PAGE analysis of freeze-dried Catcher-cVLPs before (-) or after (+) centrifugation (2 min at 16,000 g). (c) Quantification (by densitometric analysis of SDS-PAGE) of the relative amount (%) of reconstituted Catcher-cVLP, which remain in suspension after centrifugation. (d) Representative SDS-PAGE analysis of the conjugation reaction between freeze-dried Catcher-cVLPs with a tagged antigen. Samples were taken before (-) and after (+) centrifugation of the coupling mixture. Tag:Catcher conjugation results in the formation of a antigen-cVLP coupling band at 60 kDa. (e) Quantification of the antigen-cVLP coupling band from the conjugation reaction of Catcher-cVLPs with a tag-antigen. Results show the mean of samples performed in duplicate.

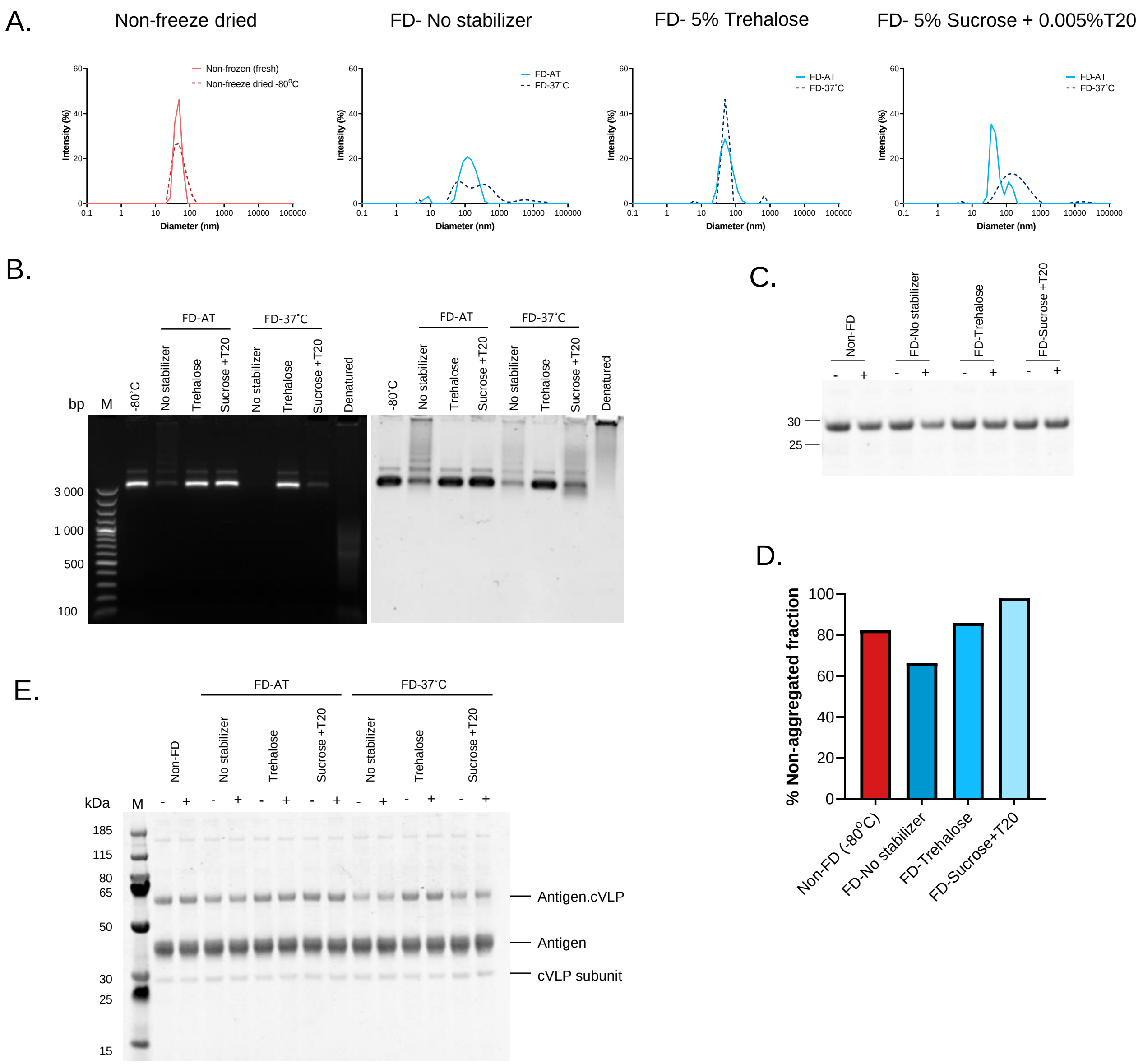


**Supplementary Figure S4. Stability of the Catcher-cVLP backbone after freeze-drying and storage for 2 months (Tris buffer).** Catcher-cVLPs formulated in a Tris buffer (pH 8.5) with no stabilizing agent, 5% trehalose or 5% sucrose + 0.005% Tween20 were freeze-dried and subsequently stored at ambient temperature (FD-AT) or 37˚C (FD-37˚C) for 2 months. The stability of reconstituted freeze-dried material was compared to a non-frozen or a frozen non-freeze dried (Non-FD (-80˚C)) reference sample. (a) DLS analysis of reference samples (red) and freeze-dried Catcher-cVLP (blue) after storage at ambient temperature (solid line) or 37˚C (dashed line). (b) Agarose gel electrophoresis stained with ethidium bromide (left) and Coomassie brilliant blue (right). A non-freeze dried reference sample was heated at 95˚C for 5 min (denatured) and run in parallel to the native samples to indicate the migration of nucleic acid relative to protein when particles have disassembled. (c) Representative reduced SDS-PAGE analysis of freeze-dried Catcher-cVLPs before (-) or after (+) centrifugation (2 min at 16,000 g). (d) Quantification (by densitometric analysis of SDS-PAGE) of the relative amount (%) of reconstituted Catcher-cVLP, which remain in suspension after centrifugation. (e) SDS-

PAGE analysis of the conjugation reaction between freeze-dried Catcher-cVLPs with a tagged antigen. Samples were taken before (-) and after (+) centrifugation of the coupling mixture. Tag:Catcher conjugation results in the formation of a antigen-cVLP coupling band at 60 kDa . M = molecular weight marker.

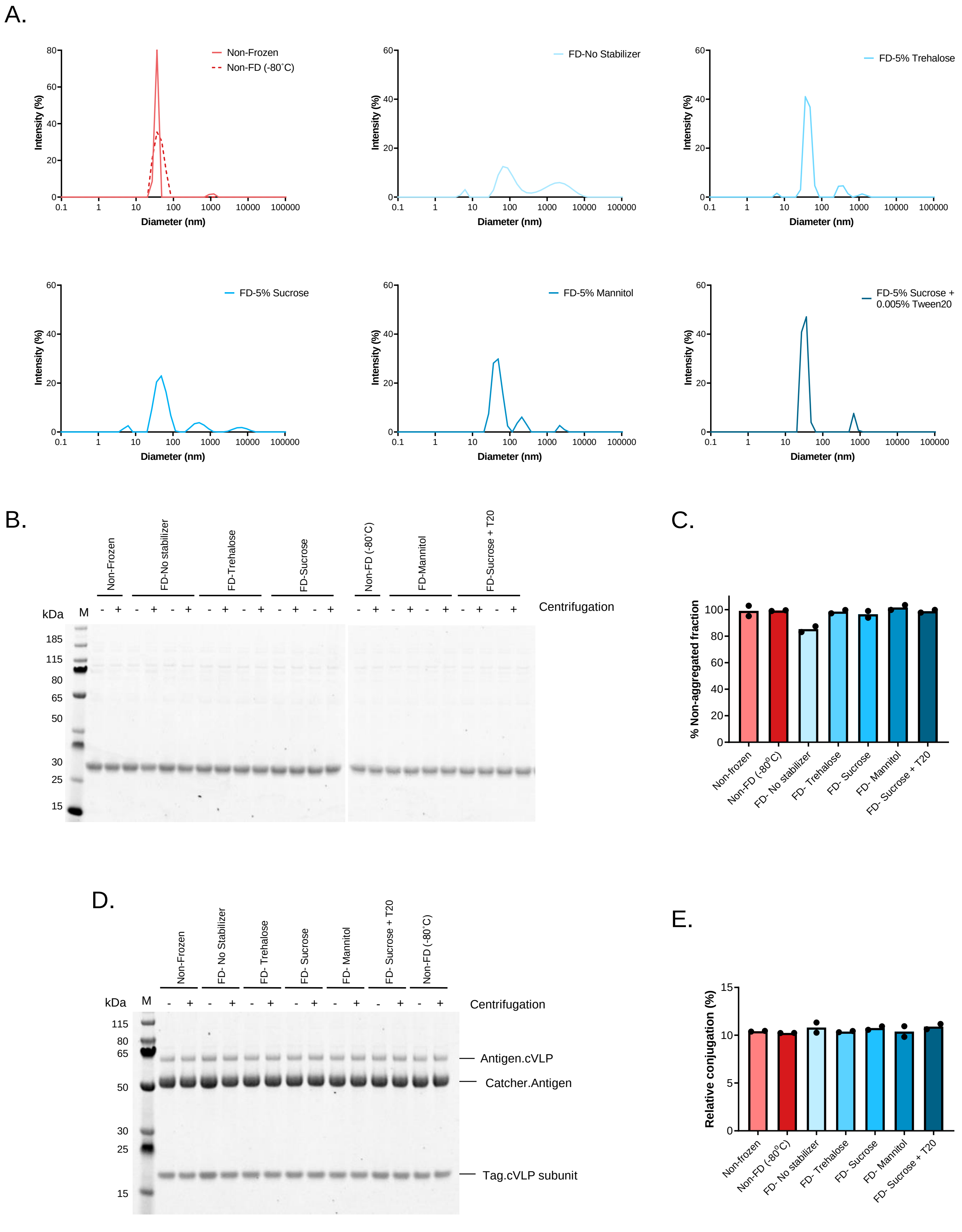


**Supplementary Figure S5. Effect of excipients on the stability of freeze-dried Tag-cVLPs in a sodium phosphate buffer (pH 7.4).** Tag-cVLPs formulated in a sodium phosphate buffer (pH 7.4) with no stabilizing agent, 5% trehalose, 5% sucrose, 5% mannitol or 5% sucrose + 0.005%

Tween20 were freeze-dried and subsequently reconstituted one day later. The stability of freeze-dried particles was compared to a non-frozen or a frozen non-freeze dried (Non-FD (-80˚C)) reference sample. (a) DLS analysis. (b) Reduced SDS-PAGE analysis of freeze-dried Tag-cVLPs before (-) or after (+) centrifugation (2 min at 16,000 g). (c) Quantification (by densitometric analysis of SDS-PAGE) of the relative amount (%) of reconstituted Tag-cVLP, which remain in suspension after centrifugation. (d) Representative SDS-PAGE analysis of the conjugation reaction between freeze-dried Tag-cVLPs with a Catcher-antigen. Samples were taken before (-) and after (+) centrifugation of the coupling mixture. Tag:catcher conjugation results in the formation of an antigen-cVLP coupling band at 64 kDa. (e) Quantification of the antigen-cVLP coupling band from the conjugation reaction of Tag-cVLPs with a Catcher-antigen. Results show the mean of samples performed in duplicate.

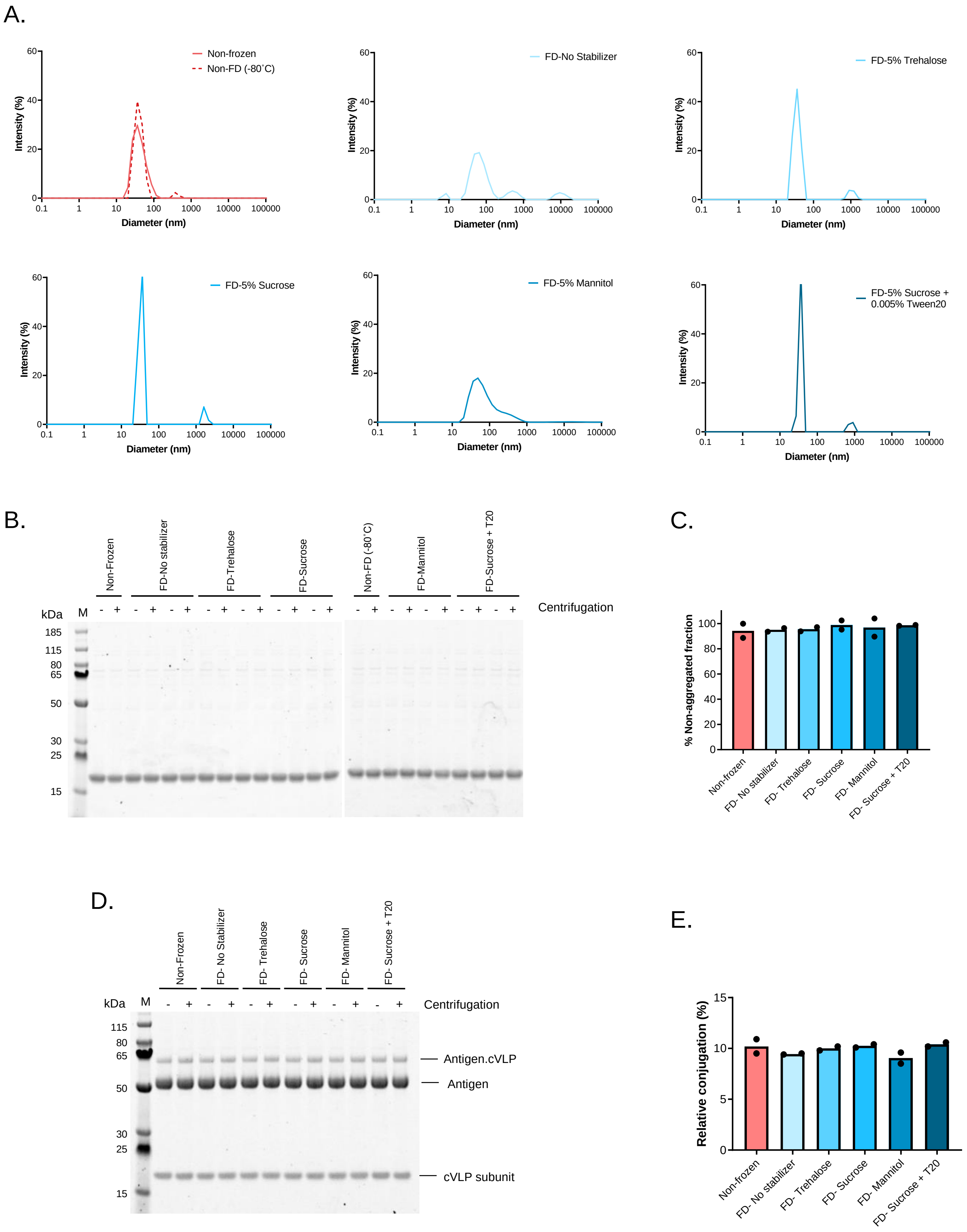


**Supplementary Figure S6. Effect of excipients on the stability of freeze-dried Tag-cVLPs in a Tris (pH 8.5).** Tag-cVLPs formulated in a Tris buffer (pH 8.5) with no stabilizing agent, 5% trehalose, 5% sucrose, 5% mannitol or 5% sucrose + 0.005% Tween20 were freeze-dried and subsequently reconstituted one day later. The stability of freeze-dried particles was

compared to a non-frozen or a frozen non-freeze dried (Non-FD (-80˚C)) reference sample. (a) DLS analysis. (b) Reduced SDS-PAGE analysis of freeze-dried Tag-cVLPs before (-) or after (+) centrifugation (2 min at 16,000 g). (c) Quantification (by densitometric analysis of SDS-PAGE) of the relative amount (%) of reconstituted Tag-cVLP, which remain in suspension after centrifugation. (d) Representative SDS-PAGE analysis of the conjugation reaction between freeze-dried Tag-cVLPs with a catcher-antigen. Samples were taken before (-) and after (+) centrifugation of the coupling mixture. Tag:Catcher conjugation results in the formation of a antigen-cVLP coupling band at 64 kDa. (e) Quantification of the antigen-cVLP coupling band from the conjugation reaction of Tag-cVLPs with a Catcher-antigen. Results show the mean of samples performed in duplicate.

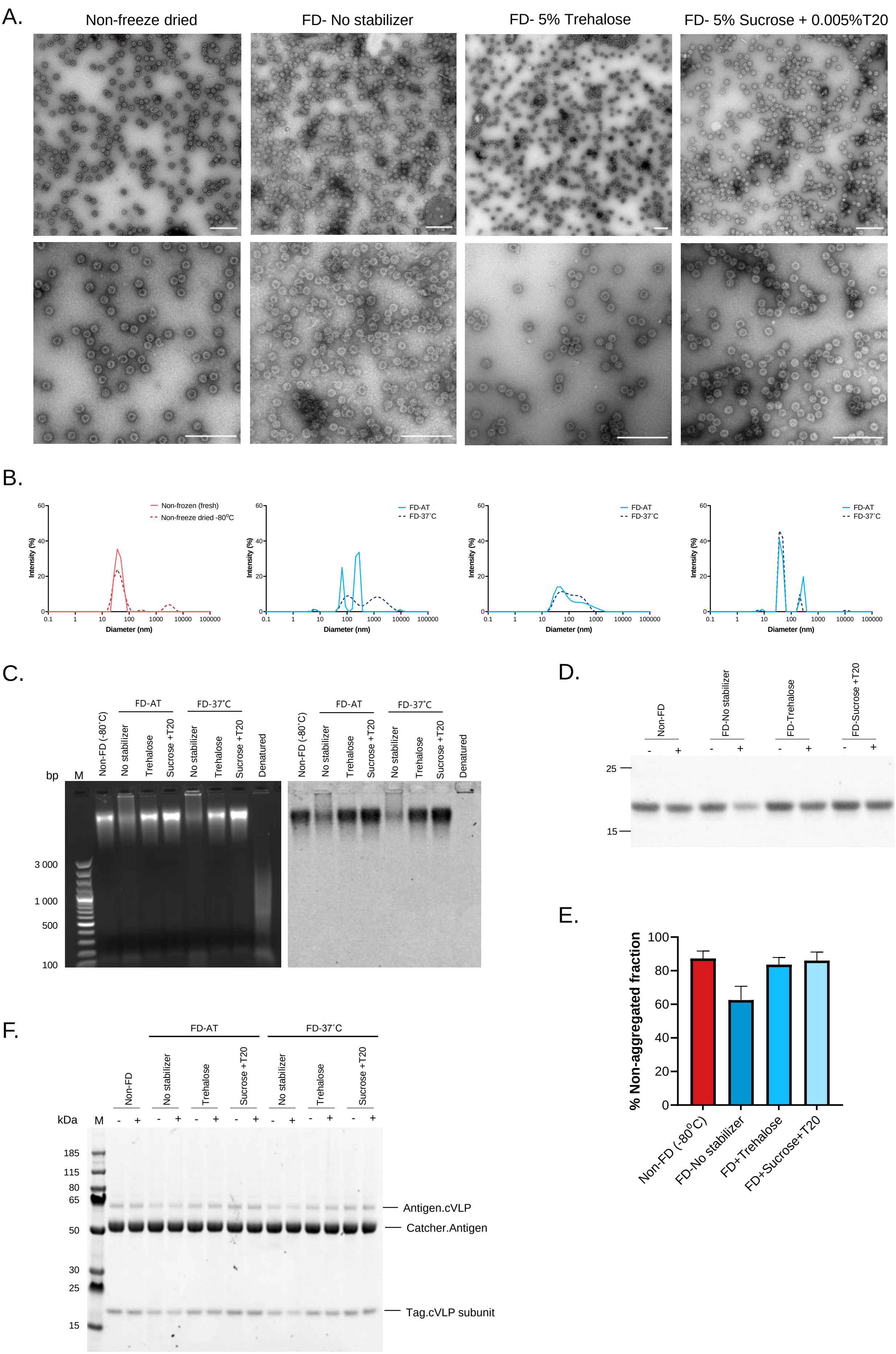
A.
Non-freeze dried
FD- No stabilizer
FD- 5% Trehalose
FD- 5% Sucrose + 0.005%T20
B.
Non-frozen (fresh)
Non-freeze dried -80°C
FD-AT
FD-37˚C
Intensity (%)
Diameter (nm)
C.
FD-AT
FD-37˚C
Non-FD (-80˚C)
No stabilizer
Trehalose
Sucrose +T20
Denatured
bp
M
3 000
1 000
500
100
D.
Non-FD
FD-No stabilizer
FD-Trehalose
FD-Sucrose +T20
25
15
E.
% Non-aggregated fraction
Non-FD (-80°C)
FD-No stabilizer
FD+Trehalose
FD+Sucrose+T20
F.
kDa
M
Non-FD
185
115
80
65
50
30
25
15
Antigen.cVLP
Catcher.Antigen
Tag.cVLP subunit

**Supplementary Figure S7. Stability of the Tag-cVLP backbone after freeze-drying and storage for 2 months (Sodium Phosphate buffer).** Tag-cVLPs formulated in a sodium phosphate buffer (pH 7.4) with no stabilizing agent, 5% trehalose or 5% sucrose + 0.005% Tween20 were freeze-dried and subsequently stored at ambient temperature (FD-AT) or 37℃ (FD-37℃) for 1 week (a) or 2 months (b-f). The stability of reconstituted freeze-dried material was compared to a non-frozen or a frozen non-freeze dried (Non-FD (-80℃)) reference sample. (a) Negative stain transmission electron microscopy (TEM) images. Scale bar represents 500nm (top panel) and 200 nm (bottom panel). (b) DLS analysis of reference samples (red) and freeze-dried Tag-cVLP (blue) after storage at ambient temperature (solid line) or 37℃ (dashed line). (c) Agarose gel electrophoresis stained with ethidium bromide (left) and Coomassie brilliant blue (right). A non-freeze dried reference sample was heated at 95℃ for 5 min (denatured) and run in parallel to the native samples. (d) Representative reduced SDS-PAGE analysis of freeze-dried Tag-cVLPs before (-) or after (+) centrifugation (2 min at 16,000 g). (e) Quantification (by densitometric analysis of SDS-PAGE) of the relative amount (%) of reconstituted Tag-cVLP, which remain in suspension after centrifugation. (f) SDS-PAGE analysis of the conjugation reaction between freeze-dried Tag-cVLPs with a Catcher-antigen. Samples were taken before (-) and after (+) centrifugation of the coupling mixture. Tag:catcher conjugation results in the formation of an antigen-cVLP coupling band at 64 kDa. M = molecular weight marker.

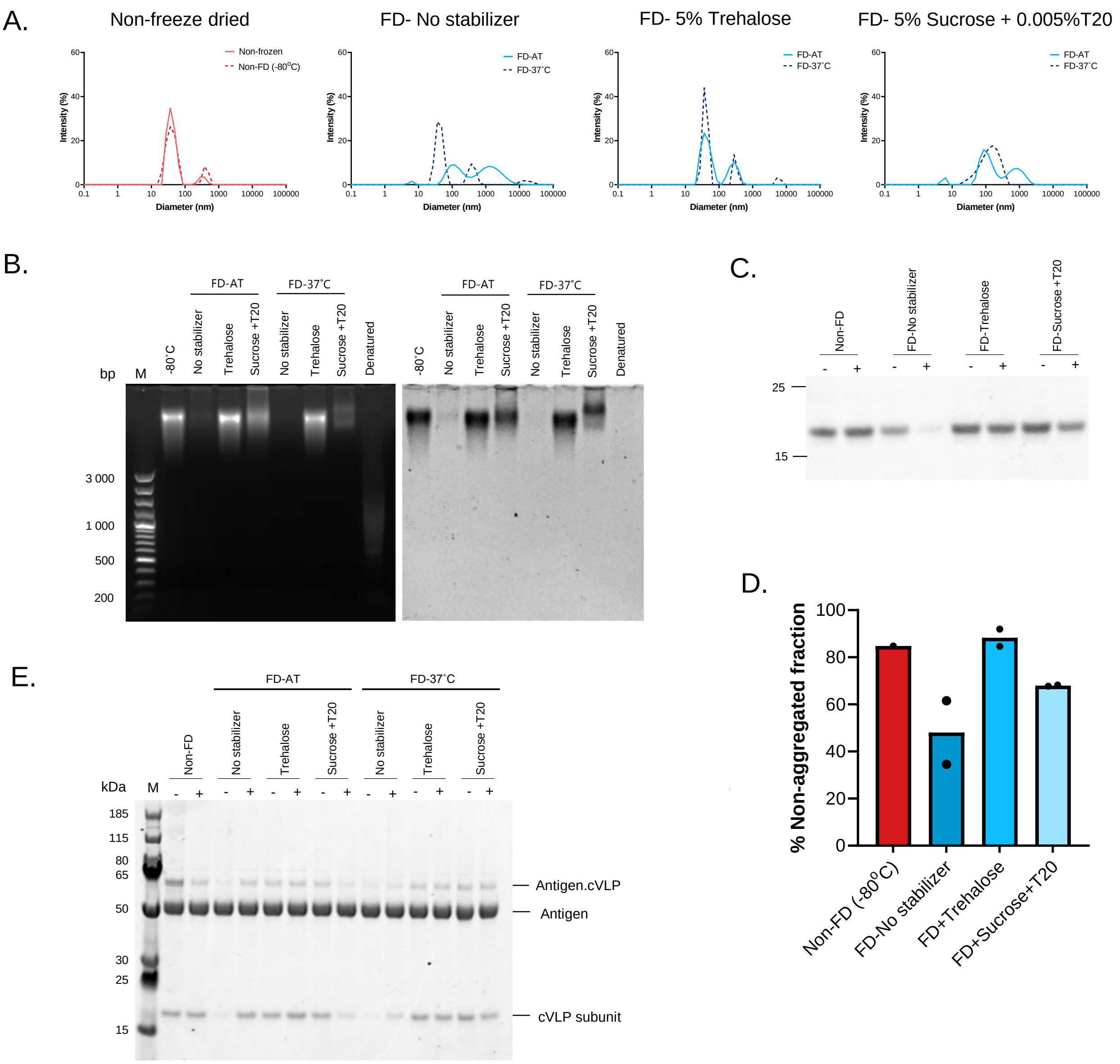


**Supplementary Figure S8. Stability of the Tag-cVLP backbone after freeze-drying and storage for 2 months (Tris buffer).** Tag-cVLPs formulated in a Tris buffer (pH 8.5) with no stabilizing agent, 5% trehalose or 5% sucrose + 0.005% Tween20 were freeze-dried and subsequently stored at ambient temperature (FD-AT) or 37℃ (FD-37℃) for 1 week (a) or 2 months (b-f). The stability of reconstituted freeze-dried material was compared to a non-frozen or a frozen non-freeze dried (Non-FD (-80℃)) reference sample. (a) DLS analysis of reference samples (red) and freeze-dried Tag-cVLP (blue) after storage at ambient temperature (solid line) or 37℃ (dashed line). (b) Agarose gel electrophoresis stained with ethidium bromide (left) and Coomassie brilliant blue (right). A non-freeze dried reference sample was heated at 95℃ for 5 min (denatured) and run in parallel to the native samples. (c) Representative reduced SDS-PAGE analysis of freeze-dried Tag-cVLPs before (-) or after (+) centrifugation (2 min at 16,000 g). (d) Quantification (by densitometric analysis of SDS-PAGE) of the relative amount (%) of reconstituted Catcher-cVLP, which remain in suspension after centrifugation. (e) SDS-PAGE analysis of the conjugation reaction between freeze-dried Tag-cVLPs with a Catcher-antigen. Samples were taken before (-) and after (+) centrifugation of the coupling mixture.

Tag:Catcher conjugation results in the formation of an antigen-cVLP coupling band at 60 kDa. M = molecular weight marker.

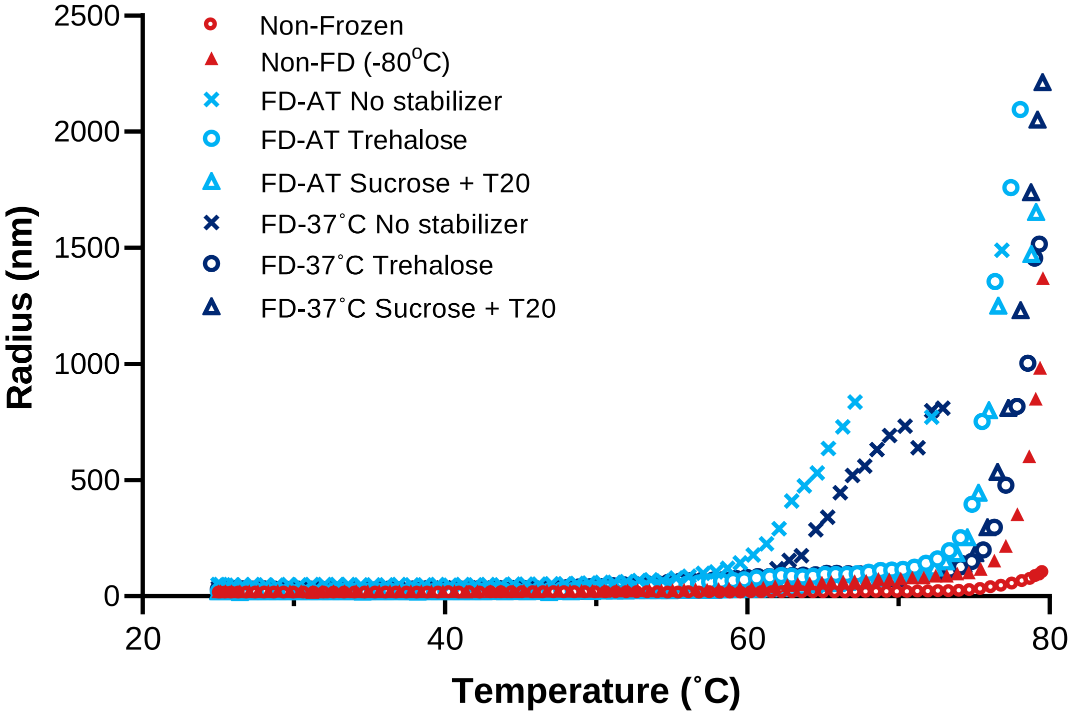


**Supplementary Figure S9. Thermal stability of freeze-dried Tag-cVLP backbone.** The hydrodynamic radius of freeze-dried Tag-cVLPs stored at ambient temperature or 37˚C for 2 months was measured by DLS as a function of temperature between 25˚C and 80˚C.

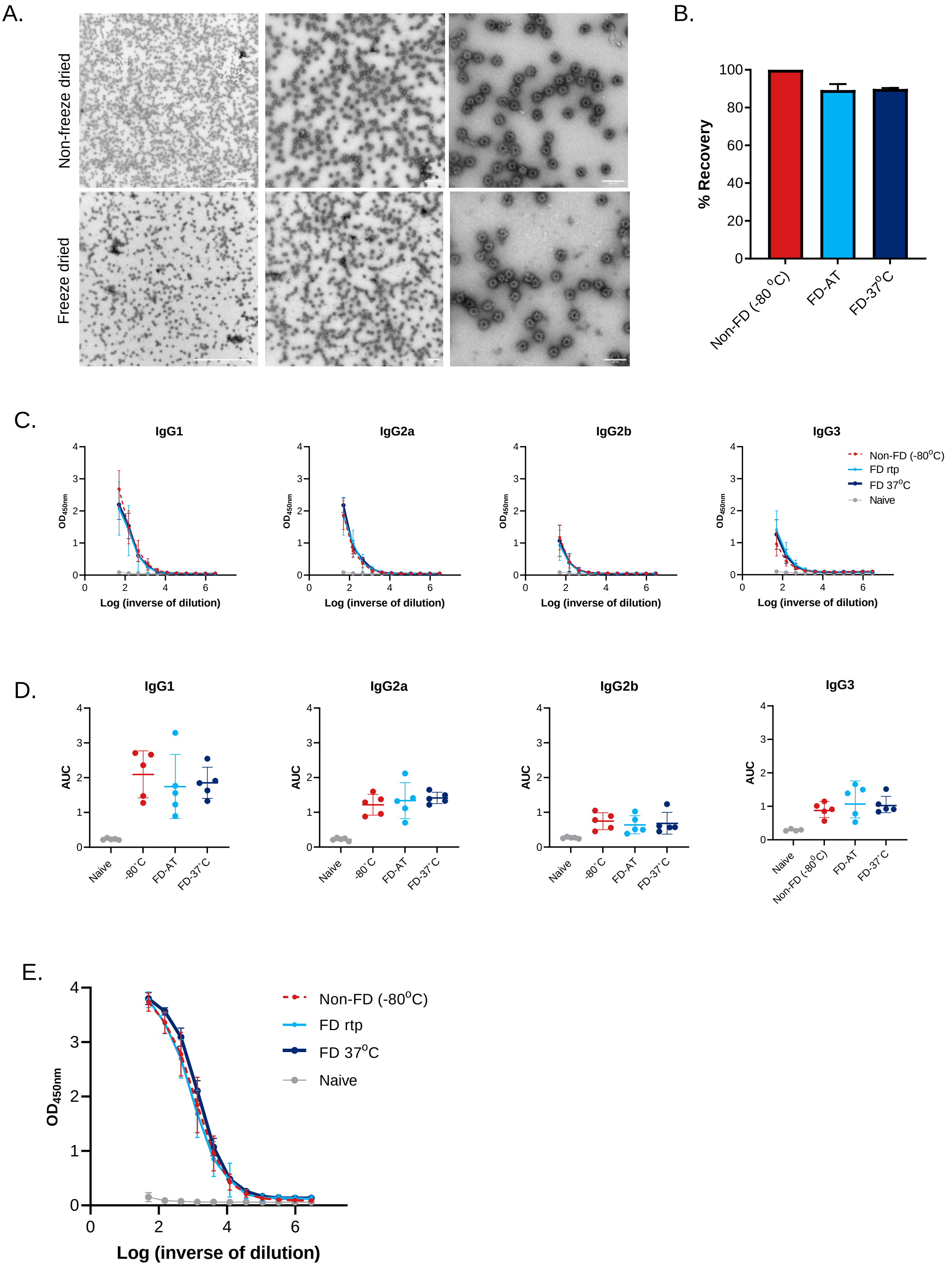


**Supplementary Figure S10. Freeze-drying of $HA_{stem}$-cVLP influenza vaccine (related to figure 2).** (a) Additional negative stain transmission electron microscopy (TEM) images of non-freeze

dried $HA_{stem}$-cVLP reference sample (top panel) and freeze-dried $HA_{stem}$-cVLP (bottom panel). (b) Percentage recovery of $HA_{stem}$-cVLP vaccine after freeze-drying, storage for 2 months and reconstitution, measured as total protein recovery relative to a non-freeze dried reference sample by BCA assay. (c) Dilution curves and (d) area under the curve titres of anti-$HA_{stem}$ IgG subclasses and (e) total IgG in the serum of mice immunized with 7 µg of non-freeze dried or freeze-dried $HA_{stem}$-cVLP vaccine. Results show the mean ± SD area under the curve titre.

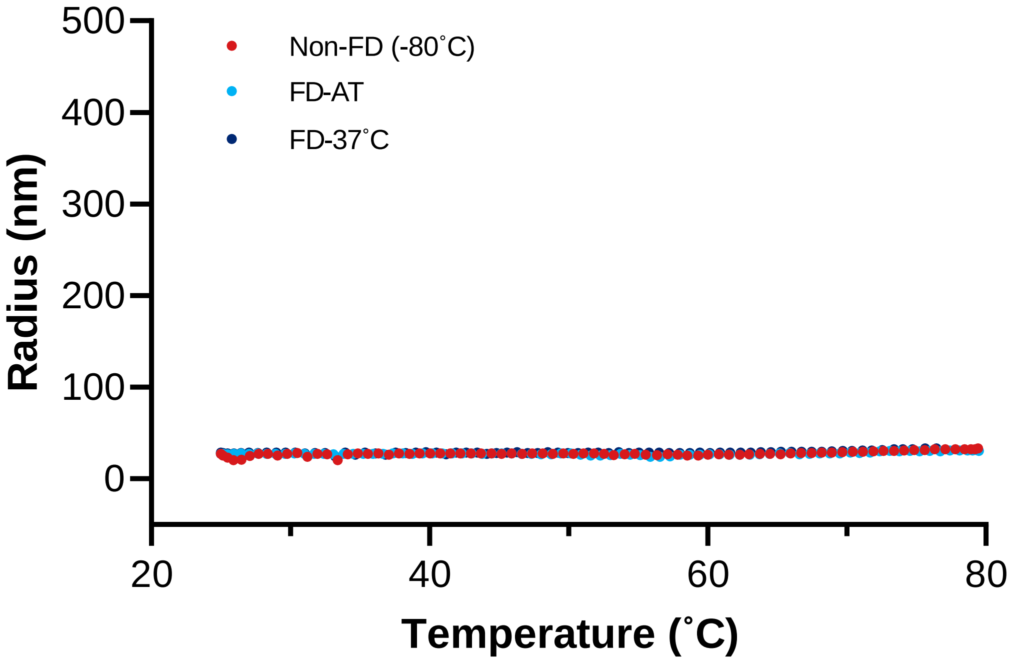


**Supplementary Figure S11. Thermal stability of freeze-dried $HA_{stem}$-cVLP influenza vaccine.** The hydrodynamic radius of freeze-dried $HA_{stem}$-cVLPs stored at ambient temperature or 37˚C for 2 months was measured by DLS as a function of temperature between 25˚C and 80˚C. Unfolding/aggregation of the particles did not occur with the temperature range.

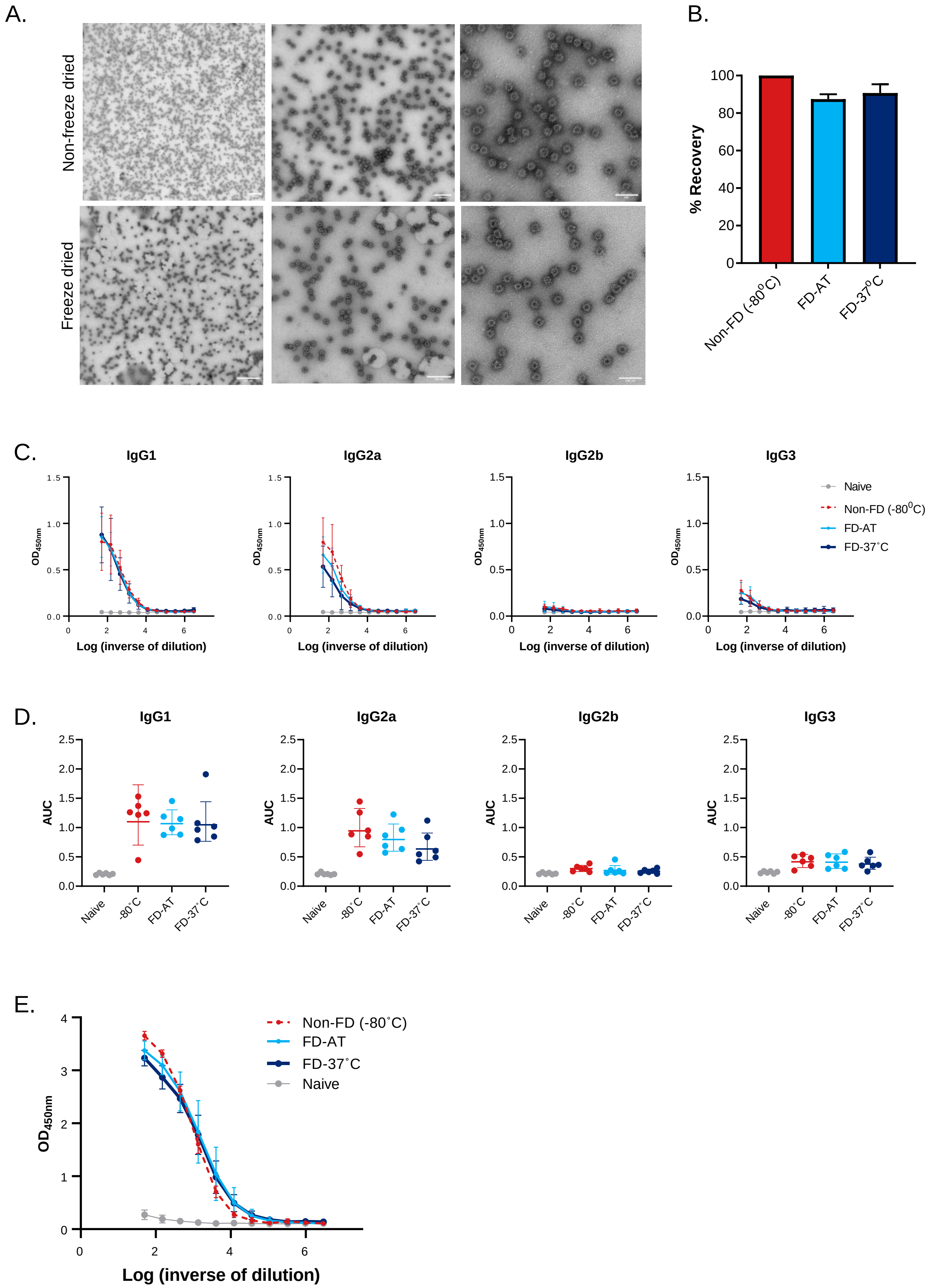


**Supplementary Figure S12. Freeze-drying of RBD-cVLP SARS-CoV-2 vaccine (related to figure 3).** (a) Additional negative stain transmission electron microscopy (TEM) images of non-freeze dried RBD-cVLP reference sample (top panel) and freeze-dried RBD-cVLP (bottom panel). (b)

Percentage recovery of RBD-cVLP vaccine after freeze-drying, storage for 2 months and reconstitution, measured as total protein recovery relative to a non-freeze dried reference sample by BCA assay. (c) Dilution curves and (d) area under the curve titres of anti-Spike IgG subclasses and (e) total IgG in the serum of mice immunized with 5 µg of non-freeze dried or freeze-dried RBD-cVLP vaccine. Results show the mean ± SD area under the curve titre.

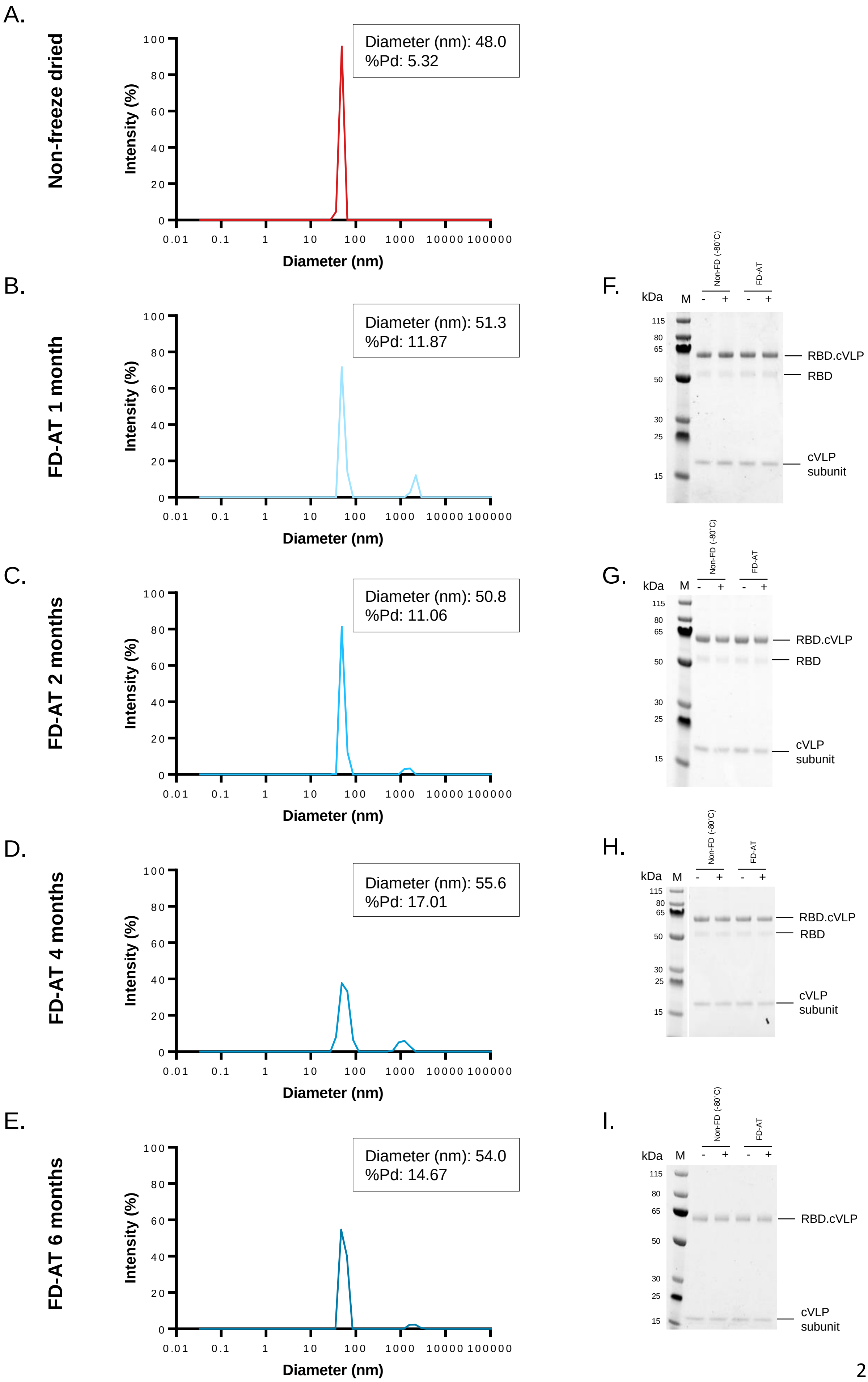

A.
Non-freeze dried
Diameter (nm): 48.0
%Pd: 5.32
B.
FD-AT 1 month
Diameter (nm): 51.3
%Pd: 11.87
C.
FD-AT 2 months
Diameter (nm): 50.8
%Pd: 11.06
D.
FD-AT 4 months
Diameter (nm): 55.6
%Pd: 17.01
E.
FD-AT 6 months
Diameter (nm): 54.0
%Pd: 14.67
Intensity (%)
Diameter (nm)
0.01 0.1 1 10 100 1000 10000 100000
0 20 40 60 80 100
F.
G.
H.
I.
Non-FD (-80˚C)
FD-AT
kDa
M
- + - +
115
80
65
50
30
25
15
RBD.cVLP
RBD
cVLP subunit

**Supplementary Figure S13. Six-month stability of freeze-dried RBD-cVLP SARS-CoV-2 vaccine at ambient temperature (related to figure 4).** Representative DLS analysis of RBD-cVLP before freeze-drying (a), or 1 month (b), 2 months (c), 4 months (d), and 6 months (e) after freeze-drying and storage at room temperature. Average hydrodynamic diameter and percentage polydispersity of the main population peak is indicated. (f-g) SDS-PAGE analysis of RBD-cVLP after 1, 2, 4 or 6 months storage respectively. Samples were taken before (-) or after (+) centrifugation (2 min 16,000 g). The 6 month samples originated from a different production batch then the other time point samples, hence the lower amount of unconjugated RBD (52 kDa) in the sample is a result of batch variance and is not a result of freeze-drying or long-term storage.

**Supplementary Table S5. Thermal stability of freeze-dried RBD-cVLP SARS CoV-2 vaccine (related to Figure 3e).** The onset temperature (temperature at which unfolding/aggregation begins) and radius of particles at onset was calculated from DLS of the RBD-cVLPs between 25˚C and 80˚C.

| Sample | Radius (nm) | Onset temperature (˚C) |
|---|---|---|
| Non FD-(80˚C) | 27.78 | 48.53 |
| FD-AT | 27.93 | 48.75 |
| FD-37˚C | 28.04 | 48.47 |
